# Multimodal emotion perception after anterior temporal lobectomy (ATL)

**DOI:** 10.3389/fnhum.2014.00275

**Published:** 2014-05-05

**Authors:** Valérie Milesi, Sezen Cekic, Julie Péron, Sascha Frühholz, Chiara Cristinzio, Margitta Seeck, Didier Grandjean

**Affiliations:** ^1^Swiss Center for Affective Sciences, University of GenevaGeneva, Switzerland; ^2^Neuroscience of Emotion and Affective Dynamics Laboratory, Department of Psychology, Faculty of Psychology and Educational Sciences, University of GenevaGeneva, Switzerland; ^3^Laboratory for Neurology and Imaging of Cognition, Department of Neurology and Department of Neuroscience, Medical School, University of GenevaGeneva, Switzerland; ^4^Epilepsy Unit, Department of Neurology, Geneva University HospitalGeneva, Switzerland

**Keywords:** amygdala, emotion perception, multimodal, prosody, facial expression

## Abstract

In the context of emotion information processing, several studies have demonstrated the involvement of the amygdala in emotion perception, for unimodal and multimodal stimuli. However, it seems that not only the amygdala, but several regions around it, may also play a major role in multimodal emotional integration. In order to investigate the contribution of these regions to multimodal emotion perception, five patients who had undergone unilateral anterior temporal lobe resection were exposed to both unimodal (vocal or visual) and audiovisual emotional and neutral stimuli. In a classic paradigm, participants were asked to rate the emotional intensity of angry, fearful, joyful, and neutral stimuli on visual analog scales. Compared with matched controls, patients exhibited impaired categorization of joyful expressions, whether the stimuli were auditory, visual, or audiovisual. Patients confused joyful faces with neutral faces, and joyful prosody with surprise. In the case of fear, unlike matched controls, patients provided lower intensity ratings for visual stimuli than for vocal and audiovisual ones. Fearful faces were frequently confused with surprised ones. When we controlled for lesion size, we no longer observed any overall difference between patients and controls in their ratings of emotional intensity on the target scales. Lesion size had the greatest effect on intensity perceptions and accuracy in the visual modality, irrespective of the type of emotion. These new findings suggest that a damaged amygdala, or a disrupted bundle between the amygdala and the ventral part of the occipital lobe, has a greater impact on emotion perception in the visual modality than it does in either the vocal or audiovisual one. We can surmise that patients are able to use the auditory information contained in multimodal stimuli to compensate for difficulty processing visually conveyed emotion.

## INTRODUCTION

The ability to decode emotional information is crucial in everyday life, allowing us to adapt our behaviors when confronted with salient information, both for survival and for social adaptiveness purposes. The emotional features of objects in the environment have been shown to bring about an increase in the neuronal response, compared with the processing of non-emotional information (for a review, see Phan et al., 2002). The role that different brain regions play in decoding emotional information appears to depend on the modality. Furthermore, research has shown that both the primary and secondary sensory regions are modulated by emotion. For example, visual extrastriate regions are modulated by emotions conveyed by facial expressions (e.g., Morris et al., 1998; Pourtois et al., 2005a; Vuilleumier and Pourtois, 2007), while temporal voice-sensitive areas have been shown to be modulated by emotional prosody (e.g., Mitchell et al., 2003; Grandjean et al., 2005; Schirmer and Kotz, 2006; Wildgruber et al., 2006; Frühholz et al., 2012).

According to Haxby’s face perception model (Haxby et al., 2000), visual information is processed along a ventral pathway leading from the primary visual cortex (V1) to the fusiform face area (FFA) and inferior temporal cortex (ITC). Face perception is sufficient to activate the FFA (see, for example, Pourtois et al., 2005a; Kanwisher and Yovel, 2006; Pourtois et al., 2010), but the activity of this structure is enhanced when the facial information is emotional (see, for example, Breiter et al., 1996; Dolan et al., 2001; Vuilleumier et al., 2001; Williams et al., 2004; Vuilleumier and Pourtois, 2007). Another structure whose activity increases when decoding emotional facial information is the amygdala (see, for example, Haxby et al., 2000; Calder and Young, 2005; Phelps and LeDoux, 2005; Adolphs, 2008). In monkeys, this structure has been shown to project to almost every step along the visual ventral pathway (Amaral et al., 2003). Human studies, meanwhile, have suggested that connectivity between the amygdala and the FFA is modulated by emotion perception (Morris et al., 1998; Dolan et al., 2001; Vuilleumier et al., 2004; Sabatinelli et al., 2005; Vuilleumier, 2005; Vuilleumier and Pourtois, 2007).

Regarding the amygdala’s role in emotion perception, the current hypothesis is that this structure detects salience, a general feature of emotion (for a discussion, see Sander et al., 2003; Armony, 2013; Pourtois et al., 2013), through reciprocal connections with the cortex (Amaral et al., 2003). Its main function is to facilitate attention and perception processing (e.g., Armony and Ledoux, 1997, 1999; Whalen, 1998; Vuilleumier et al., 2001) without explicit voluntary attention (for a review, see Vuilleumier and Pourtois, 2007). According to Ledoux’s (2007) model, the amygdala’s output is directed both to regions that modulate bodily responses (via the endocrine system related to the autonomic system), and to the primary and associative cortices. These encompass regions modulated by emotion such as in the extrastriate visual system, the FFA for face perception, and the voice area in the superior temporal gyrus (STG; including the primary auditory region).

Further insight into emotional face perception and its subcortical bases has been provided by studies of patients with lesions of the amygdala. More specifically, studies have assessed patients with temporal lobe epilepsy whose lesions are linked either to the epileptogenic disease itself or else to its surgical treatment (see, for example, Cristinzio et al., 2007). These studies included patients with congenital or acquired diseases resulting in bilateral lesions, and patients with unilateral epilepsy arising from mesial temporal sclerosis who had undergone lobectomy with amygdalectomy. Patients with bilateral damage have been found to display impaired fearful face perception (Adolphs et al., 1994, 1995; Young et al., 1995; Calder et al., 1996; Broks et al., 1998) and deficits in the perception of surprise and anger (Adolphs et al., 1994). Unilateral lesions have yielded either no differences (Adolphs et al., 1995; prior surgery, Batut et al., 2006) or else a deficit for patients with right-sided lesions covering either a range of emotions (Anderson et al., 2000; Adolphs and Tranel, 2004) or solely fearful faces (prior surgery, Meletti et al., 2003). Palermo et al. (2010) found that both left- and right-lesion groups exhibited a deficit in fear intensity perception, but the left-lesion group was more impaired for fear detection. Anterior temporal lobectomy with amygdalectomy is generally expected to affect the perceived intensity of facial emotional expressions. The functional explanation for this is a lack of modulation by the amygdala of the ventral visual processing network and, more specifically in the case of emotional faces, of the FFA.

In addition to visual emotional information, the amygdala has been shown to be associated with different responses to emotional vocalizations. According to Schirmer et al. (2012), the processing of auditory information takes place along three streams in the temporal lobe: a posterior stream passing through the posterior part of the superior temporal sulcus (pSTS) for sound embodiment; a ventral stream directed toward the middle temporal gyrus (MTG) for concept processing; and an anterior stream extending as far as the temporal pole (TmP) for the perceptual domain (i.e., semantic processing). Another specificity of emotional vocalization perception is the hemispheric specificity modeled by Schirmer and Kotz (2006). In their model, the left temporal lobe has a higher temporal resolution for processing information than the right hemisphere, and is more involved in linguistic signal processing (segmental information), with suprasegmental analysis taking place in the right hemisphere. The amygdala has been shown to be modulated by emotional vocalizations, including onomatopoeia (e.g., Morris et al., 1999; Fecteau et al., 2007; Plichta et al., 2011), and emotional prosody consisting either of pseudowords (e.g., Grandjean et al., 2005; Sander et al., 2005; Frühholz and Grandjean, 2012, 2013), or of words and sentences (e.g., Ethofer et al., 2006, 2009; Wiethoff et al., 2009).

In contrast to research on emotional face perception, studies of auditory emotion processing in patients with bilateral amygdala lesions have produced divergent results. Some have failed to find any effect at all on emotion recognition (semantically neutral sentences: Adolphs and Tranel, 1999; names and onomatopoeia: Anderson and Phelps, 1998). Others have reported either a general impairment (counting sequences: Brierley et al., 2004) or specific impairments for fear (semantically neutral sentences: Scott et al., 1997; non-verbal vocalizations: Dellacherie et al., 2011), surprise (Dellacherie et al., 2011), anger (Scott et al., 1997), or sadness perception (musical excerpt: Gosselin et al., 2007). There is a similar divergence for unilateral lesions, with either no effects (Adolphs and Tranel, 1999; Adolphs et al., 2001) or a specific impairment for fear (counting sequences: Brierley et al., 2004; meaningless words: Sprengelmeyer et al., 2010; non-verbal vocalizations: Dellacherie et al., 2011). To sum up current knowledge about auditory emotion processing, there is a strong hypothesis about right hemispheric involvement for emotional prosody. The amygdala appears to be involved in prosody perception, but may also be sensitive to the proximal context of the stimulus presentation (for a discussion, see Frühholz and Grandjean, 2013).

In the case of face-voice emotion integration, studies featuring audiovisual emotional stimuli have replicated the response facilitation effect at the behavioral level, namely an increase in perceptual sensitivity and reduced reaction times (e.g., Massaro and Egan, 1996; De Gelder and Vroomen, 2000; Dolan et al., 2001; Kreifelts et al., 2007), that has already been demonstrated in non-emotional studies (e.g., Miller, 1982; Schröger and Widmann, 1998). Responsibility for the behavioral improvement has been mainly attributed to various cortical substrates, including the left MTG (e.g., Pourtois et al., 2005b), the posterior STG (pSTG; e.g., Ethofer et al., 2006; Kreifelts et al., 2007), and, interestingly, the amydala, either bilaterally (e.g., Klasen et al., 2011) or the left side (e.g., Dolan et al., 2001; Ethofer et al., 2006; Müller et al., 2012). Animal studies have yielded a more detailed multimodal model, with different levels of integration. For instance, a rhinal cortex lesion, as opposed to a direct lesion of the amygdala, is sufficient to disrupt associative mechanisms (Goulet and Murray, 2001). Meanwhile, a comparison of the roles of the perirhinal cortex (PRC) and the pSTS led Taylor et al. (2006) to suggest that the pSTS plays a presemantic integration role, while the PRC integrates higher level conceptual representations.

In summary, studies of the amygdala’s modal specificity have reported impairments in patients with temporal lobectomy or specific amygdalectomy for faces and either voices (Scott et al., 1997; Sprengelmeyer et al., 1999; Brierley et al., 2004) or emotion in music (Gosselin et al., 2007, 2011). However, some patients seem to have a specific deficit for visual emotional stimuli (Adolphs et al., 1994, 2001; Anderson and Phelps, 1998; Adolphs and Tranel, 1999). Discrepancies between studies have been explained by a number of different factors, including the date of epilepsy onset (e.g., McClelland et al., 2006), the nature and context of the stimuli (e.g., face presentation duration; Graham et al., 2007; Palermo et al., 2010). The fear specificity of amygdala processing has also been strongly called into question (for a discussion, see Cahill et al., 1999; Murray, 2007; Morrison and Salzman, 2010). To the best of our knowledge, however, the role of lesion size has not been taken in account thus far.

Our aim in the present study was to test whether the categorization and intensity perception of unimodal (i.e., either visual or non-verbal auditory emotional stimuli), as opposed to bimodal (i.e., audiovisual) emotional stimuli is modified in patients who have undergone unilateral temporal anterior lobectomy with amygdalectomy. The impact of anterior temporal lobe ablation is assumed to differ with modality. Regarding the auditory network, above and beyond the absence of voice area modulations owing to amygdala resection, Schirmer et al. (2012) suggests that the anterior temporal lobe is more involved in semantic processing, representing the final temporal step before the processing shifts to the frontal regions associated with emotion evaluation. We would therefore expect disruption of this input to have an impact on categorization, with patients making more mistakes or confusing more items than matched controls. For the visual modality, we would expect to find the same kind of deficit, stemming from the lack of emotion-related modulation of visual cortical input. Finally, for audiovisual material, we would expect to observe either a better preserved ability for correct detection and perceived intensity, if an intact pSTS and a more dorsal pathway toward the frontal lobe are sufficient to integrate audiovisual information, or no improvement because of the PRC lesion.

Participants rated the intensity of brief onomatopoeic vocalizations produced by actors (Bänziger et al., 2012) and animated synthetic faces (Roesch et al., 2011) on visual analog scales. At the group level, we expected the patients to have a higher error rate than controls when it came to identifying unimodal emotional stimuli. This has been shown to be the case in the visual modality for fearful faces (bilateral lesion: Adolphs et al., 1994, 1995; Young et al., 1995; Calder et al., 1996; Broks et al., 1998; unilateral lesion: Anderson et al., 2000; McClelland et al., 2006), and in the auditory modality for both fearful voices (bilateral lesion: Scott et al., 1997; Adolphs and Tranel, 2004; unilateral lesion: Scott et al., 1997; Brierley et al., 2004; Sprengelmeyer et al., 2010; Dellacherie et al., 2011) and angry voices (bilateral lesion: Scott et al., 1997). For the audiovisual stimuli, we expected to observe a higher error rate for fear identification, arising from the combined effects of the unimodal deficits in each modality. Regarding intensity perception, we expected to observe similar patterns, even after controlling for the extent of the lesion along the ventral pathway. Finally, we investigated the effects of lesion size on emotion recognition. We predicted that perception of emotion intensity would be modulated by the size of the lesion, with more extensive lesions resulting in impairment at different levels of information processing. We developed an additional hypothesis to explain the discrepant findings of previous studies.

## MATERIALS AND METHODS

### PARTICIPANTS

**FIGURE 1:**
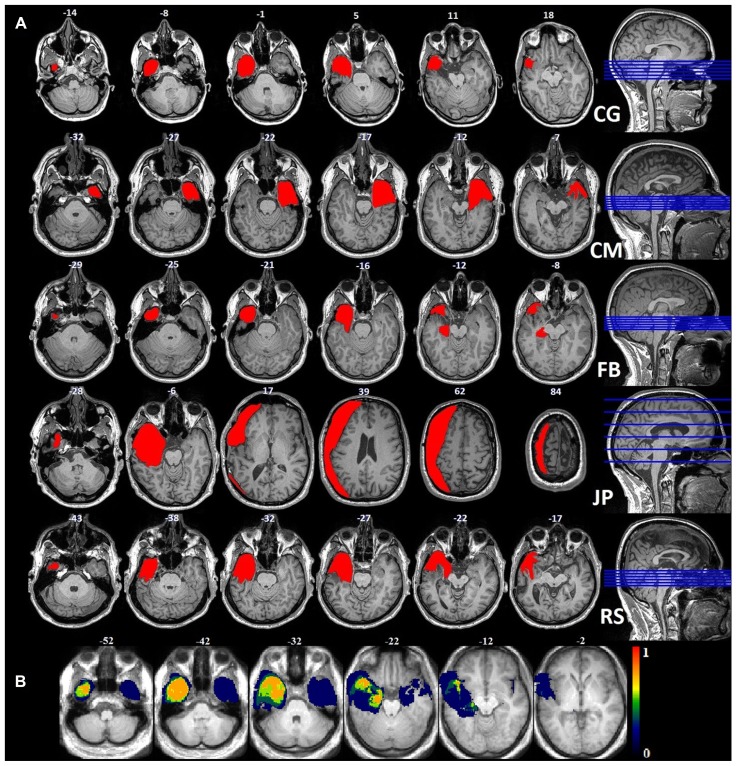
**(A)** Anatomical images of the lesions for each patient: each lesion was delineated manually on the axial plane and corrected using the coronal plane. **(B)** Probability map for the normalized lesion size.

We recruited five patients who had undergone unilateral anteromedial temporal lobectomy together with the unilateral removal of the amygdala. One patient (JP) had a lesion that extended to the occipital and posterior parietal lobes. The surgery had been performed to control the patients’ medically intractable seizures (see **Figure 1** for the location and extent of their lesions): four on the left side (FB, 23 years old; CG, 37 years old; JP, 45 years old; and RS, 62 years old) and one on the right (CM, 31 years old). CG was the only woman in the patient group, and FB the only left-handed patient. Controls were recruited via local advertisements: 12 were matched with FB, CM, and CG for sex, handedness, and age; six with JP; and three with RS (see **Table 1** for a summary and **Table 2** for a detailed description of each patient). Patients did not exhibit any gnosis deficit in their respective neuropsychological tests. The study was approved by the local ethics committee, and all the participants gave their written informed consent. The controls received financial compensation (CHF 15) for taking part in the experiment.

**Table 1:** Participants.

Patient	Lateralization	Age (years)	Sex	Lesion (vx) (volume, normalized volume)	Controls *n*	Mean age (SD) of controls
CG	Right-handed	37	Female	Left (31′564, 40′683)	12	33.42 (3.63)
CM	Right-handed	31	Male	Right (36′836, 48′833)	12	32.83 (2.48)
FB	Left-handed	23	Male	Left (21′596, 30′208)	12	22.08 (1.88)
JP	Right-handed	45	Male	Left (434′284, 113′254)	6	45.17 (2.32)
RS	Right-handed	62	Male	Left (44′011, 53′920)	3	60 (4)

**Table 2:** Patient description.

Patient	Years since lobectomy	Age at epilepsy onset	Comorbidity	Diagnosis
CG	5	12 years	–	Left temporal partial complex epilepsy with hippocampal sclerosis
CM	6	11 years	Anxiety	Left hemiplegia with right hemisphere hypoplasia
FB	10	–	–	Major left hippocampal sclerosis
JP	11	5 years	Depressed feeling (at time of surgery)	Left temporal epilepsy
RS	7	6 months	–	Left hippocampal sclerosis

### LESION DELIMITATION AND DESCRIPTION

In order to compute the lesion size of each patient, anatomical images were segmented and normalized using a unified segmentation approach (Ashburner and Friston, 2005) together with the Clinical toolbox^1^. Because of the cost function masking purpose (Andersen et al., 2010), lesion masks drawn on the patients’ anatomical scans were included in the brain segmentation. Structural images and lesion masks were normalized to MNI space with the DARTEL toolbox, using individual flow fields, which were estimated on the basis of the segmented gray (GM) and white matter (WM) tissue classes. The normalized lesion masks were used to calculate the lesion size for each patient in standard space.

CG had a left anterior temporal lesion with an intact inferior temporal gyrus (ITG) and lateral occipitotemporal gyrus (LOTG). The lesion area included the periamygdaloid cortex (PAM), entorhinal cortex (Ent), medial occipitotemporal gyrus (MOTG), inferior part of the hippocampus (Hi), parahippocampal gyrus (PHG), and amygdala, and ended in the lateral anterior portion of the temporal lobe, in the MTG and TmP.

CM had a right anterior temporal lesion extending to the middle and ventromedial part of the temporal lobe, including the inferior temporal pole (ITmP), ITG, Ent, PAM, PRC, amygdala, inferior Hi, STG, anterior fusiform gyrus (FuG), and rhinal sulcus. In the posterior part of the lesion, the PPo (planum polare), STG and STS were intact.

FB had a left anterior temporal lesion that included the TmP, MTG, MOTG, Ent, Hi, PAM, amygdala, anterior STG, and posterior temporal cortex (PTe). The lesion ended in two separate tails: one in the lateral anterior part of the temporal lobe, the other in the medial part.

JP had an extended left lateral resection including the temporal, frontal, parietal, and occipital lobes. The temporal part included the TmP, MTG, Ent, MOTG, ITG, PTe, anterior STG, and PHG. The frontal part included the lateral inferior and superior frontal gyri, precentral gyrus and postcentral gyrus. Finally, part of the lateral superior posterior occipital gyrus had been removed, but the FFA was intact.

RS had a left anterior temporal lesion encompassing the TmP, STG, MTG, MOTG, ITG, FuG, amygdala, anterior Hi, Ent, PAM, FuG and anterior PHG. It ended in the lateral anterior part. See **Figure 1** for visual descriptions of the patients’ brain damage.

### STIMULI AND PROCEDURE

Non-verbal auditory expressions were drawn from the validated Geneva Multimodal Emotion Portrayal (GEMEP) corpus (Bänziger et al., 2012). We selected angry, joyful, and fearful non-verbal sounds (“ah”) produced by two male and two female actors, on the basis of the recognition rate established in a previous pilot study. For the neutral stimuli, we chose the most neutrally rated vocal expressions produced by the same actors (neutrality rating: *M* = 26.5, SD = 15.67), and the fundamental frequency was flattened using Praat (Boersma and Weenink, 2011). Sounds were cut and/or stretched to achieve a duration of 1 s (mean duration before time stretch = 0.92 s, SD = 0.30 s) with SoundForge^2^, and 0.025 s fade-ins and fade-outs were included using Audacity^3^. The dynamic faces were created with FACSGen (Roesch et al., 2011), which allows for the parametric manipulation of 3D emotional facial expressions according to the Facial Action Coding System (Ekman and Friesen, 1978). They were selected on the basis of results of a previous study in which participants assessed the gender and believability of each avatar (Roesch et al., 2011). The lips were animated to match the intensity contour of each different sound for both unimodal visual and audiovisual items. The action units (AUs) for each emotion began at 0.25 s and ended at 0.75 s after onset, with their apex at 0.5 s (100% intensity). VirtualDub^4^ was used to generate the image sequences and to combine the voiced sounds with them at a rate of 26 frames per second (the final image was a dark screen).

After signing the consent form, participants completed the behavioral inhibition system (BIS)/behavioral approach system (BAS) scales and the state trait anxiety inventory (STAI) on a web interface. They then rated the intensity of 216 items in unimodal [auditory (A), or visual (V)] and audiovisual (AV; congruent: same information in both modalities; incongruent: one modality emotional, the other neutral) conditions. The unimodal and congruent audiovisual stimuli could either express the emotions of anger, fear, or joy, or be neutral (control condition). Each condition (modality, emotion, or congruency) was repeated 12 times. Items were presented using E-Prime (standard v2.08.90^5^) in a pseudorandomized order to avoid repetition of the same stimulus (i.e., synthetic face or actor’s voice) or condition. The participants gave their answers by clicking on a continuous line between *Not intense* and *Very intense* for six different emotions (disgust, joy, anger, surprise, fear, sadness), plus neutral. In each trial, they could provide ratings on one or more scales. At the end of the experiment, they completed a debriefing questionnaire.

### STATISTICAL ANALYSIS

Since multiple intensity scales were used to collect the answers, our data mostly contained zero ratings. To assess the interactions, we therefore ran a zero-inflated mixed model on congruent trials only, using the glmmADMB package for R^6^. This allowed the excess zeroes and remaining values to be modeled as binomial responses, and modeled the distribution as a generalized linear model (GLM) following a negative binomial distribution. Main effects were tested for group (control vs. patient), modality (audio, visual, audiovisual), and emotion (anger, fear, or joy, plus neutral). Contrasts were performed to test specific hypotheses.

The first hypothesis we tested was a group effect for a specific emotion on the target scale (e.g., fearful item ratings on the fear scale) for each modality (A, V, AV). Sex, age, and normalized lesion size were added as control variables. Participant and stimulus ID were added as random effects. A different model was run for each of the three emotions, plus neutral. Second, four different models, one for each emotion, plus neutral, were tested in order to compare the impact of the three different modalities in each group. For instance, for angry item ratings on the anger scale, the modalities were tested in pairs (AV-A, AV-V, A-V) for the patient group, and individually for the control group. For this second set of models, we added the same control and random variables as for the first model. The third model was run to investigate the lateralization effect of the lesion for a specific modality and a specific emotion, controlling for handedness, age, and sex, and with random effect variables for participant ID and stimulus ID. Owing to the limited size of our patient sample, this comparison was of a purely descriptive and exploratory nature. In order to test whether the effects we found in the different modalities were perceptual or emotional, we ran a complementary analysis to compare emotional versus neutral items in each modality and each group, adding age, sex, and normalized lesion size as control variables, and participant ID and stimulus ID as random effects. Intergroup effects were also tested for emotional versus neutral items in each modality (A, AV, V), with the same control variables. Finally, we tested the impact of lesion size by including the number of voxels in a separate linear model for each emotion and each modality. In this final set of models, random effect variables (participant ID and stimulus ID) were added.

## RESULTS

### CATEGORICAL RESPONSES

Participants could rate the intensity of each item on six different scales (anger, disgust, fear, surprise, joy, sadness, and neutral). For each item, we identified the scale with the highest rating, and calculated a proportional corrected score for each participant (Heberlein et al., 2004; Dellacherie et al., 2011), by looking at how many other members of the participant’s group (patient or control) had given the same response. This score could range from 0, meaning that nobody else in the group had chosen the same scale, to 1, meaning that everyone in the group had chosen the same scale. This type of correction is used to weight labeling errors, bearing in mind that some errors are more correct than others. For instance, it is easier to confuse visual fear and surprise (see, for example, Etcoff and Magee, 1992) than it is to confuse fear and anger, as the first two expressions share a number of AUs. For vocal expressions, confusion is also possible, but between different pairs of emotions (see, for example, Banse and Scherer, 1996; Belin et al., 2008; Bänziger et al., 2009).

**FIGURE 2:**
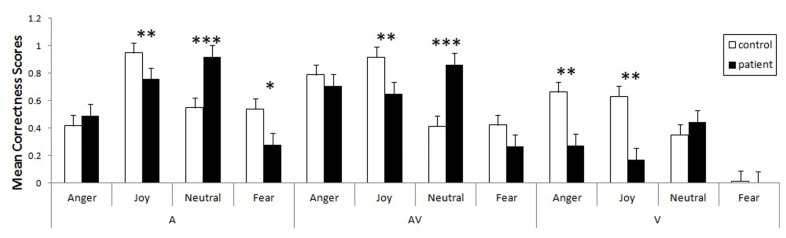
Mean proportional corrected scores for patients and controls, taking modality and emotion into account (bars represent the standard error of the mean, *p < 0.05, **p < 0.005, ***p < 0.001).

Using these corrected scores, we looked for possible differences between the two groups. As our data violated the assumptions of homoscedasticity and normal distribution, we ran non-parametric tests for multiple groups. In order to pinpoint differences between the groups within a specific emotion in a specific modality, we used the Kruskal–Wallis test, calculating *z* scores and *p* values corrected for multiple comparisons of mean ranks (*z*′). These multiple comparisons are summarized in **Figure 2**. The control group was more accurate than the patient group in recognizing joy, whether it was expressed vocally (*z*′ = 3.02, *p* < 0.005), visually (*z*′ = 3.17, *p* < 0.005), or bimodally (*z*′ = 3.19, *p* < 0.005). Greater accuracy within the control group was also observed for visual anger (*z*′ = 2.99, *p* < 0.005), vocal fear (*z*′ = 2.78, *p* < 0.01) and - marginally - visual (*z*′ = 1.69, *p* = 0.08) and bimodal fear (*z*′ = 1.89, *p* = 0.058). Finally, a reverse group effect was observed for the neutral vocal (*z*′ = 3.64, *p* < 0.001) and audiovisual (*z*′ = 3.64, *p* < 0.001) stimuli.

Finally, we tested the impact of lesion size on the corrected hit rate for emotion recognition. We ran supplementary analyses using a GLM to test this effect with the modality (A, AV, V) and emotion (anger, joy, fear, neutral) factors, and added the normalized lesion size as a covariate. The control variables were age, sex, and lateralization. We observed a significant linear relationship between normalized lesion size and corrected hit score for visual anger (*z* = -2.91, *p* < 0.005), visual joy (*z* = -2.37, *p* < 0.05) and visual neutral stimuli (*z* = -3.52, *p* < 0.001). All the linear regressions were negative, meaning that the more extensive the lesion, the lower the corrected score. We observed no such effect for fear in the visual modality, as patients did not recognize this emotion (their corrected score was equal to 0), confusing it with surprise.

### INTENSITY PERCEPTION

**FIGURE 3:**
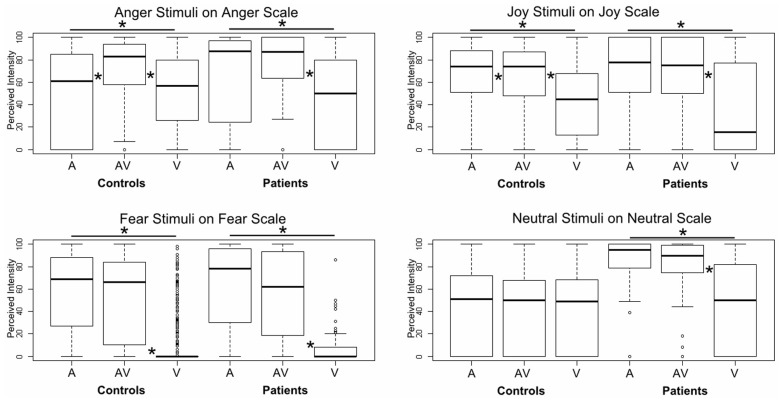
**Boxplot of GLM results for intensity ratings of each emotion on the corresponding target scale.** Each box corresponds to a specific modality (A: auditory, AV: audiovisual, V: visual) and a specific group (patients vs. controls). The difference between A and AV for the controls is almost invisible, as zero values data were included in the plot. *Indicates a significant difference between modalities (pairwise).

Using a GLM, we first compared the two groups on each specific emotion in each specific modality, controlling for sex, age, and normalized lesion size, and adding participant and stimulus ID as random effects. No significant results were observed, even for the fear items. However, when we ran pairwise comparisons of the modalities for a specific emotion on its target scale and for a specific group, we did observe significant effects, especially for the three emotions (see **Figure 3**). Patients provided higher intensity ratings of audiovisual versus unimodal visual information for angry (*z* = -4.14, *p* < 0.001), joyful (*z* = -6.14, *p* < 0.001), fearful (*z* = -8.45, *p* < 0.001), and neutral (*z* = -5.61, *p* < 0.001) items. They also provided higher intensity ratings of auditory versus visual information for the same emotions (anger: *z* = -4.14, *p* < 0.001; joy: *z* = -6.14, *p* < 0.001; fear: *z* = -8.45, *p* < 0.001; neutral: *z* = -5.61, *p* < 0.001). The differences between audiovisual and unimodal auditory information were not significant for any of the emotions (*p* > 0.15). In the control group, a slightly different pattern emerged for anger and joy. Anger was given a higher intensity rating in the audiovisual condition than in either the auditory (*z* = -3.27, *p* < 0.001) or visual (*z* = -10.93, *p* < 0.001) condition, and a higher rating in the auditory condition than in the visual one (*z* = -6.94, *p* < 0.001). For joy, audiovisual information was perceived of as more intense than visual information (*z* = -12.69, *p* < 0.001), but auditory information was given a higher intensity rating than both audiovisual information (*z* = 3.07, *p* < 0.005) and visual information (*z* = -15.58, *p* < 0.001). Finally, fear stimuli were rated as more intense in the audiovisual modality than in the visual one (*z* = -11.74, *p* < 0.001), and also more intense in the auditory modality than in the visual one (*z* = -12.33, *p* < 0.001). No significant differences were observed between the modalities for neutral stimuli (*p* > 0.4).

In order to ascertain whether the results were perceptual or emotional, we tested another model contrasting emotional versus neutral stimuli for each group and each modality (A, V, AV). Controls rated emotional auditory items as more intense than neutral auditory items (*z =* 5.61, *p* < 0.001), and this was also the case for audiovisual information (*z =* 5.15, *p* < 0.001). By contrast, the patients provided higher intensity ratings for neutral items than they did for emotional items in the auditory (*z* = -2.64, *p* < 0.01) and audiovisual (*z* = -2.42, *p* < 0.05) modalities. In the visual modality, patients (*z* = -3.56, *p* < 0.001) and controls (*z* = -8.40, *p* < 0.001) alike gave higher intensity ratings for neutral items than for emotional ones. When we compared the two groups on emotional and neutral items in each modality, we found that the patients rated the intensity of the neutral items more highly than the controls did in the auditory modality (*z* = 2.24, *p* < 0.025). For the audiovisual modality, the effect was only marginal (*z* = 1.86, *p* = 0.062).

### INTENSITY PERCEPTION AND LESION EFFECT

**FIGURE 4:**
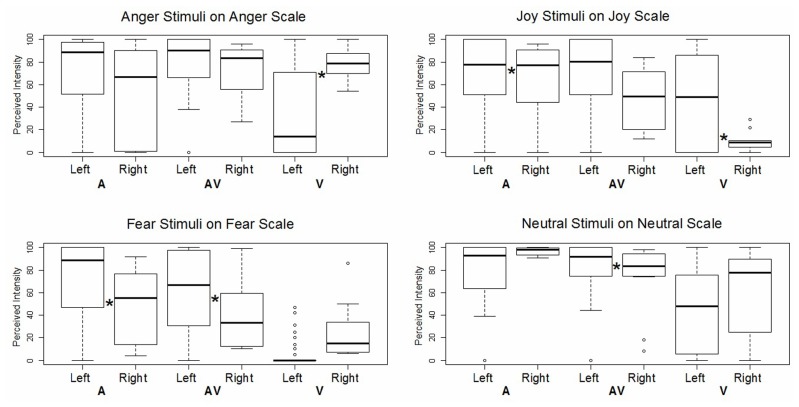
**Boxplot of GLM results for intensity ratings of each emotion on the corresponding target scale.** Each box corresponds to a specific modality (A: auditory, AV: audiovisual, V: visual) and a specific patient subgroup (left lesion vs. right lesion). *Indicates a significant difference between left and right lesion conditions.

We then assessed the impact of lesion lateralization for each specific emotion in each specific modality. In this GLM analysis, we compared the patients’ ratings on the target scale according to the side of their lesion, controlling for handedness, age, and sex, and adding participant and stimulus ID as random effects (see **Figure 4**). The patient with a right lesion was found to provide higher intensity ratings than the patients with left lesions, but only for angry faces (*z* = -4.36, *p* < 0.001) and auditory joy (*z* = -3.23, *p* < 0.005). All other significant effects concerned the opposite relationship, namely, the patients with left lesions rated the items as more intense than the patient with a right lesion did. This was the case for visual joy (*z* = 3.19, *p* < 0.005), auditory fear (*z* = 8.29, *p* < 0.001), audiovisual fear (*z* = 8.23, *p* < 0.001), and audiovisual neutral items (*z* = 3.67, *p* < 0.001).

When we added the normalized lesion size as a covariate and compared the interactions of perceived intensity and modality for a specific emotion on the target scale, we observed a massive effect in the visual modality across all emotions: the larger the lesion, the less intensely the patients perceived visual anger (*z* = -2.90, *p* < 0.005), visual joy (*z* = -2.79, *p* < 0.005), and visual fear (*z* = -2.96, *p* < 0.005). Neutral visual stimuli, however, failed to reach significance (*p* > 0.15). This relationship also held good for audiovisual joy (*z* = -2.15, *p* < 0.051), but no significant effects were observed either for other audiovisual expressions (angry, fearful or neutral), or for auditory stimuli (*p* > 0.15).

## DISCUSSION

### CATEGORICAL RESPONSES

Our main goal was to investigate the relationship between emotion and modality, comparing patients who had undergone unilateral anterior temporal lobectomy and amydalectomy with a matched control group. Overall, proportional corrected scores revealed that patients detected joy less accurately across all modalities, in contrast to previous studies postulating that impairments are restricted to negatively valenced stimuli (e.g., Brierley et al., 2004). In addition, the patients displayed deficits for auditory fear and visual anger. The massive effect we observed for decoding joy has several possible explanations. First, this effect could be associated with the amount of information needed for accurate decoding. For instance, Graham et al. (2007) reported that patients were impaired in categorizing emotional faces when these were only presented for a limited duration. In the auditory domain, timing is also a crucial feature for prosody decoding. In healthy individuals, researchers have shown that there is a positive correlation between the duration of the sound and the correct recognition of the vocal stimulus (Pollack et al., 1960; Cornew et al., 2010; Pell and Kotz, 2011). Furthermore, happy prosody needs a duration of at least 1 s to be decoded accurately (Pell and Kotz, 2011), and our stimuli included 0.25 s fade-ins and fade-outs, thus reducing the amount of available information and its actual duration. The second explanation also concerns a lack of information. In the visual items, the lips were animated to match the intensity contour of each vocal stimulus, even in the unimodal visual condition. As a result, this manipulation may have had an impact on emotion recognition because the information needed to detect a smile was masked by the movement of the lips accompanying the vocalization. More specifically, the visual cues in the mouth region that are needed to detect joy (AU 12 – lip corner puller) and anger (AUs 0 – upper lip raise, 17 – chin raise, 23 – lip funnel, 24 – lip press) were less visible, and thus less salient. Although we expected fear perception accuracy to be poorer among patients than among controls across all the modalities, we found that it was only diminished for auditory stimuli, indicating that unilateral amygdala damage is not sufficient to impair fear recognition in the visual domain. Numerical differences in the confusion matrix (**Table 3**) suggest that the lack of an effect for visual information stemmed from the fact that fearful faces and faces expressing surprise were confused by both patients (62%) and controls (71%). This confusion between fear and surprise at the visual level is easily explained by the proximity of the AUs used to produce these emotional expressions. In actual fact, they differ by only two AUs: one in the brow region (AU 4 – brow lowerer), the other around the mouth (AU 20 – lip stretcher).

**Table 3:** Confusion matrix.

Target emotion												
	Auditory information				Audiovisual information				Visual information			
Responses	Anger	Joy	Neutral	Fear	Anger	Joy	Neutral	Fear	Anger	Joy	Neutral	Fear
**Controls**
Anger	**62**	0	1	10	**88**	1	3	3	**81**	2	2	1
Disgust	9	1	5	4	4	0	4	5	8	2	3	1
Joy	3	**96**	2	3	0	**94**	2	2	0	**79**	4	2
Neutral	1	1	**75**	1	1	1	**64**	2	1	11	**59**	11
Fear	21	0	1	**70**	5	1	2	**61**	1	2	3	**13**
Surprise	2	1	16	10	1	2	24	23	8	4	28	71
Ambivalent	2	1	0	2	1	1	1	4	1	0	1	1
**Patients**
Anger	**68**	0	0	10	**77**	3	3	3	**53**	2	3	1
Disgust	5	0	0	2	7	2	0	0	10	7	2	2
Joy	5	**83**	0	0	0	**79**	2	2	0	**40**	5	0
Neutral	2	5	**95**	3	2	10	**90**	8	10	39	**64**	27
Fear	7	0	2	**50**	7	0	2	**48**	15	2	16	**3**
Surprise	5	12	2	27	0	3	3	37	10	5	7	62
Ambivalent	8	0	1	8	7	3	0	2	2	5	3	5

*Percentage of responses for each target emotion in each modality on the six rating scales. Bold values indicate the percentage of correct responses for an emotion on the target scale. The “ambivalent” response category corresponds to high intensity ratings on more than one scale for the same emotion.*

Interestingly, the patients were more accurate than controls in their detection of neutral expressions in both the auditory and audiovisual modalities. In this experiment, controls may have been biased toward emotional stimuli, in that 75% of items contained emotional information. They were therefore more driven to search for emotional cues in the faces. Assuming that emotion detection plays a functional role, we can surmise that it is less detrimental to identify an object as emotional, than to miss information that could indicate a threat. One can also argue that the patients’ emotion detection networks were less activated (expressed behaviorally by emotional blunting) by emotional stimuli, meaning that a neutral item was more likely to be perceived of as non-emotional.

### INTENSITY PERCEPTION

First, controls and patients alike provided lower intensity ratings for visual emotional items than for auditory or audiovisual ones. More specifically, the control group rated visual angry, joyful, and fearful items as significantly less intense, while the patient group gave significantly lower intensity ratings for all the visual items (both emotional and neutral), when lesion size was taken into account. This less intense perception of visual stimuli could be explained by the differing nature of the auditory (real human voices) and visual (synthetic faces created with FACSGen) items. Nevertheless, the control group exhibited specific patterns of intensity perception for auditory and audiovisual items, depending on the emotion. In the case of anger, audiovisual items were perceived of as being more intense than unimodal auditory ones. This could be interpreted as an increase in the perceived potential threat, driven by the redundant information in the bimodal condition, as we are hard-wired to attribute particular importance to threat-related signals in order to avoid danger more effectively (Marsh et al., 2005). For joy, we observed the opposite pattern, in that auditory joyful items were rated as more intense than audiovisual items. Finally, there was no difference between the intensity ratings provided for auditory and audiovisual fear items, either in the control group or in the patient group. It seems, therefore, that anterior temporal lobe lesions disrupt the processing not just of fear-related stimuli, but also of other emotions in the visual modality. An additional analysis comparing emotional and neutral items showed that patients produced higher intensity ratings for neutral items than for emotional ones, regardless of modality. This effect across modalities lends further weight to the assumption of emotional blunting among these patients. When we compared the groups on emotional and neutral items for each modality, we found that differences only showed up in the auditory and audiovisual modalities, with higher ratings for neutral items provided by patients compared with controls. It is not entirely clear whether the lesions alone were responsible for this effect or whether a more general dysfunction of the epileptic brain was to blame, although the correlations between lesion size and emotional judgments suggest that the lesions themselves had an impact, beyond a general epileptic effect.

The present data indicate that the anterior temporal lobe plays a variety of roles, depending on the modality. First, patients exhibited a greater deficit in intensity perception for the visual modality as a linear function of lesion size for all emotional expressions. This result highlights an important role of this region at the end of the ventral visual pathway, regardless of the nature of the emotional information. Second, modality had an impact on the ratings provided by the controls for specific emotions. Anger, for instance, was perceived of as more intense in the auditory modality than in the audiovisual or visual ones, while joy was perceived of as more intense in the audiovisual modality than in the two unimodal ones. This emotional modality preference has already been flagged up by Bänziger et al. (2009). Until now, however, it has never been observed in patients. It could be linked to the deficit in the visual pathway mentioned earlier, as no differences were observed between the unimodal auditory condition and the audiovisual one, suggesting that the disruption of the visual processing channel meant that the processing focus had to be switched to the auditory modality. We can therefore hypothesize that our patients’ audiovisual processing was impaired as a consequence of a lack of input from the visual pathway toward the anterior temporal lobe. Crossmodal integration in the PRC, an associative area in the anterior temporal lobe that has been highlighted in both animal (e.g., Goulet and Murray, 2001) and human (e.g., Taylor et al., 2006) studies, may therefore play a major role in audiovisual integration.

### INTENSITY PERCEPTION AND LESION EFFECT

We expected the patient with right amygdala damage to exhibit a greater deficit than those with left damage, given that emotion perception decoding appears to be right-lateralized (e.g., Adolphs, 2002; Schirmer and Kotz, 2006). Different deficit patterns were observed, however, depending on emotion and modality. The patient with a right temporal lesion displayed a deficit in auditory and audiovisual fear perception, along with a deficit in visual joy perception, while the left-lesion patients rated joyful prosody and angry visual expressions as less intense. These two last emotions can be seen as *approach* emotions, and BAS scores have been shown to correlate with activity in the left hemisphere (Harmon-Jones and Allen, 1997; Coan and Allen, 2003).

In addition to the lateralization effect, results highlighted a major impact of lesion size, mainly for the recognition and intensity ratings of visual emotional items. This massive visual impairment could be explained by the impact of the resection on part of the visual “what” (ventral) pathway: the absence or disruption of this component of the visual pathway system may have had a greater effect because of the reduced cues for determining expressions in the visual stimuli (i.e., masking by lip movements matched with vocalizations). Based on prior research with animals (Ungerleider and Mishkin, 1982), Catani and Thiebaut de Schotten (2008) showed, using diffusion tensor imaging, that the inferior longitudinal fasciculus, a ventral associative bundle, connects the occipital and temporal lobes (more specifically, the visual areas) to the amygdala. Given that lesion size particularly seemed to affect the visual modality in our study, we can surmise that a compensatory mechanism was at work, whereby the lack of discriminating information in a specific modality triggered a shift toward another modality (see, for example, Bavelier and Hirshorn, 2010).

### LIMITATIONS

The first caveat regarding our experiment concerns the small number of patients, and the fact that only one patient had undergone a right anterior temporal resection, while another had a larger resection. However, the discrepancy between the number of patients and the number of controls did not impede our statistical analysis, owing to our choice of model and the fact that we tested every model excluding Patients JP or CM to see if we observed any change, which was not the case. The more important point to take into consideration is the difference between the visual and auditory information. The sounds were taken from the GEMEP database, which features real human voices. By contrast, the visual stimuli were non-natural faces (i.e., avatars), and this difference could account for the increased difficulty in labeling the expressions, even though they matched the Ekman coding system (see FACSGen; Roesch et al., 2011).

## CONCLUSION

The results revealed a visual deficit in the perceived intensity of emotional stimuli. This deficit was explained by lesion size, in that the larger the lesion, the lower the intensity ratings for the visual items. This could be caused by disruption to the visual pathway connecting the occipital lobe and the amygdala, but further investigation is needed to test this hypothesis. Furthermore, emotional blunting effects may also have played a part, given that the neutral expressions were given higher intensity ratings by patients than by controls. It would be useful to determine whether the absence of audiovisual enhancement in the patients’ perception can be accounted for solely by the amygdala or whether the absence of the PRC, an area that has already been identified as an integrating area in both animals (Goulet and Murray, 2001) and humans (Taylor et al., 2006), is also an important factor.

## Conflict of Interest Statement

The authors declare that the research was conducted in the absence of any commercial or financial relationships that could be construed as a potential conflict of interest.

## Footnotes

1 http://www.mccauslandcenter.sc.edu/CRNL/clinical-toolbox

2 http://www.sonycreativesoftware.com/soundforge

3 http://audacity.sourceforge.net

4 http://www.virtualdub.org/

5 http://www.pstnet.com/eprime.cfm

6 http://www.r-project.org/
